# Pandemic Performance Measures of Resilience for Healthcare and Education in the Netherlands

**DOI:** 10.1002/hpm.3943

**Published:** 2025-05-20

**Authors:** Sophie Hadjisotiriou, Tom H. Oreel, Vincent A. W. J. Marchau, Hubert P. L. M. Korzilius, Jannie Coenen, Vittorio Nespeca, Etiënne A. J. A. Rouwette, Vítor V. Vasconcelos, Rick Quax, Heiman F. L. Wertheim, Marcel G. M. Olde Rikkert

**Affiliations:** ^1^ Geriatrics Department Radboud University Medical Centre Nijmegen the Netherlands; ^2^ Methods Radboud University Institute of Management Research Nijmegen the Netherlands; ^3^ Computational Science Lab University of Amsterdam Informatics Institute Amsterdam the Netherlands; ^4^ POLDER Center, Institute for Advanced Study University of Amsterdam Amsterdam the Netherlands; ^5^ Centre for Urban Mental Health University of Amsterdam Amsterdam the Netherlands; ^6^ Department of Medical Microbiology Radboud University Medical Centre Nijmegen the Netherlands

**Keywords:** education management, health care management, pandemic resilience, performance measures, policymaking, resilience, system resilience, system thinking

## Abstract

During the COVID‐19 pandemic, policymakers focused on improving health outcomes and safeguarding healthcare availability, which have led to negative consequences for other societal systems that persist today. The impact of these policies on health and non‐healthcare systems depends on the resilience of these systems, that is, the capability of a system to maintain functioning during crises by using its adaptive capacity and transformative response. Policymaking during the COVID‐19 pandemic might have benefitted from considering the resilience of non‐healthcare societal systems and the impact of policy choices on these systems. However, so far, the development of resilience indicators for complex systems and their application in a pandemic context remains undervalued. Therefore, in this paper, we developed performance measures for the resilience of healthcare and education as showcases for pandemic policymaking. We applied a disaster management model (COPEWELL) to both the healthcare and educational system in the Netherlands. An initial list of performance measures for each system was established based on their national quality registries. To safeguard face and content validity actors ranked these measures for each system, resulting in five performance measures for each. The healthcare resilience measures cover healthcare performance both inside and outside hospitals, and the education resilience measures apply to primary, secondary schools, and higher education. Assessing the added value of multisystem policymaking using such resilience measures is a next step to be taken.

**Summary**

- Pandemic policymaking could have used resilience assessment of various systems.
- Developed pandemic performance measures of resilience for healthcare and education.
- Pandemic monitoring in healthcare and education needs different time scales.
- Measures aid policymaking to monitor pandemic effects on social functioning.

## 1 Introduction

The COVID‐19 pandemic brought unprecedented challenges to policymaking worldwide. In September 2021, the Organization for Economic Cooperation and Development (OECD) emphasised that the COVID‐19 pandemic acutely illustrated the necessity to improve the healthcare systems' resilience [1]. Wernli et al. (2021) discuss the need to build societal resilience towards future pandemics to minimise the impact of such crises [2]. Policymakers faced many uncertainties regarding the most strategic and beneficial policies. Decisions were often reactive, responding to upcoming public health and healthcare information. They prioritised population health and the healthcare system, overlooking the (long‐term) effects on other (non‐healthcare) societal systems. All systems have been affected directly by the viral spread and indirectly by policy measures like lockdowns. As such, policies were often less effective or ineffective in reducing COVID‐19's societal impact and had unintended consequences due to unexpected interactions between systems. This led to many services in the other societal systems not being accessible to large populations, with persistent effects (e.g., school closures with long‐term learning delays, specifically for students from socially vulnerable families [3, 4, 5, 6, 7]).

Despite this, as far as we know, resilience has not been systematically used in the management or policymaking of the COVID‐19 pandemic [8]. Assessing the resilience of these systems could have guided policymakers in implementing more effective and efficient policies across society. To operationalise resilience assessment of systems in policymaking, indicators for resilience are required. International efforts have emerged to address this gap. Initiatives such as OECD reports on health system resilience, the incorporation of resilience into national health system performance assessment frameworks (e.g., Belgium), and the Partnership for Health System Sustainability and Resilience (EU Expert Advisory Group) indicate growing recognition of its importance [9, 10, 11, 12, 13].

Resilience of systems has many definitions. Resilience is the ability of a system, such as healthcare or society, to absorb shocks and recover after a crisis. In other words, the ability to resist and adapt to the impact of a crisis. This involves recovering to a past state or finding a new state of equilibrium through an adaptive, transformative, or restorative response [14, 15]. The resilience concept is currently employed in many disciplines, including ecology, psychology, healthcare, engineering, and economy [16, 17], among others, to inform policymaking. In disaster management, resilience frameworks have been used to predict and preventively improve a community's resilience. The Composite of Post‐Event Well‐Being (COPEWELL) model developed by Links et al. for disasters of any kind, is a conceptual framework and system dynamics‐based model to strengthen resilience in the United States, before, during, and after a disaster [16]. This model predicts how the availability of public services may develop throughout different disaster scenarios. Additionally, a self‐assessment toolkit was developed for community stakeholders and policymakers to evaluate and strengthen the resilience of their societal functions (e.g., communication, economy, education, food, and water) to disasters [18]. The COPEWELL rubric outlines a four‐phase co‐development process for resilience assessment, which includes (1) defining a self‐assessment model, (2) developing and refining assessment tools with stakeholders, (3) pilot testing in a community, and (4) finalising and integrating the full rubric through feedback and revisions. The COPEWELL model has been used for disaster preparedness on several spatial scales in the United States, showing its adaptability [19]. Other resilience frameworks exist, such as the EnRICH community resilience framework [20]. The COPEWELL model is distinct in its system dynamics base.

Ongoing research emphasizes that resilience indicators are needed, but so far, they have primarily been suggested for healthcare related to the pandemic [12, 13]. Blauer (2022) discusses the importance of community resilience in public health, such as the COVID‐19 pandemic [21]. Since resilience frameworks have not been applied to pandemics, including the COVID‐19 pandemic, nor to other societal domains during a pandemic, and pandemic decision‐making shares many similarities with disaster management, this study explores the applicability and validity of the COPEWELL framework for monitoring resilience in pandemic policymaking and its applicability beyond the U.S.

First, this requires knowledge of the preconditions for broader applicability of COPEWELL resilience tools. In policymaking, operationalizing resilience involves using performance measures to quantify and monitor system functioning and state. We define performance measures of resilience as performance metrics that, when analysed over time, enable the discovery of dynamic changes in the resilience of a system. To achieve this, performance measures should be well‐defined, operationalized, automated, and tested for validity, reliability, comprehension, and translatable in policymaking terms.

Therefore, this paper aims to define and test a structured approach to develop, assess, and validate performance measures for resilience monitoring across relevant societal domains for and during a pandemic using the first and second phases of the COPEWELL rubric as a guiding principle [18]. These phases focus on identifying and validating resilience indicators, which are essential before progressing to pilot testing and implementation. Alongside international initiatives that emphasize resilience in health system performance, this study contributes by developing and validating resilience performance measures specifically for pandemic policymaking. This approach includes developing a list of performance measures based on national registries and validation through a questionnaire by actors within the field. We report on this approach for the Dutch healthcare and educational systems and discuss their further use in monitoring societal resilience in healthcare and education.

## 2 Methodology

**FIGURE 1:**
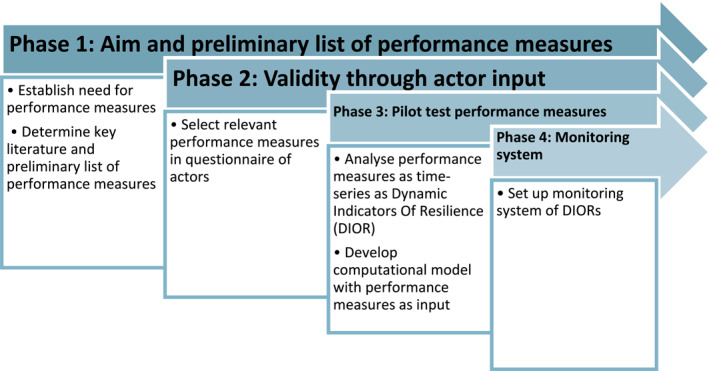
Development of performance measures to quantify the resilience of societal systems.

Based on development phases one and two of the four mentioned in the COPEWELL Process‐rubric, we aim to define and test a development and assessment method for (pandemic) resilience measures [18], by starting with the healthcare and education domains across the Netherlands (see Figure 1, phases 1 and 2).

### 2.1 Phase 1: Aim and a Preliminary List of Performance Measures of Resilience

The COVID‐19 pandemic significantly impacted healthcare and education in different ways. The healthcare system immediately felt the impact of the pandemic, while the effects on the educational system became increasingly apparent as time progressed. As many countries did, hospitals received a large influx of patients, creating resource shortages and overwhelming workloads for healthcare providers. This led to a lack of long‐term healthcare provision for standard care (e.g., diabetes, oncology, and children with disabilities) [22, 23, 24]. Simultaneously, frequent school closures aimed at limiting transmission had long‐term effects on educational outcomes and the wellbeing of pupils and students [25, 26, 27, 28, 29].

An initial search on Google Scholar for documents evaluating the healthcare system led to a focus on European health indicators and health consumer reports. Specifically, the 2018 ‘European Health Consumer Index (EHCI)’ and ‘Health at a Glance: Europe 2020—State of Health in the EU Cycle’ were identified as possible sources for establishing a preliminary list of resilience performance measures [30, 31]. However, deriving performance measures from these sources proved to be complex and overly specific, showing little evidence to be linked directly to the resilience of the healthcare system. As a result, sources of the pandemic in the Netherlands were sought and the decision was made to focus on a national source, namely the ‘Monitor Availability of Healthcare’ by the Dutch Healthcare Authority (NZa) [32]. This official document, published monthly, addressed the COVID‐19 pandemic's impact on acute healthcare facility availability in the Netherlands, emphasising its central role in public service. Given the document's significance, its multiple updates throughout the pandemic, its specific application to the Dutch context, and the data availability for these measurements, this report was selected as the main source for defining healthcare performance measures. The research group incorporated all government‐tracked and reported metrics into the questionnaire, alongside their corresponding measurement descriptions as outlined in the report. This resulted in the inclusion of 25 data‐driven and resilience‐related measures in the healthcare questionnaire, ensuring alignment with the study's focus on resilience monitoring.

Building on prior experience in defining the healthcare performance measures, the focus was quickly directed to Dutch reports of the educational system during the pandemic to establish the preliminary performance measures of resilience for education. These educational resilience measures were based on cohort research conducted by the Netherlands' directing body of educational investigations, and the 2022 national education report by the Inspection of the Educational System in the Netherlands [33, 34]. Defining performance measures from these documents proved to be more abstract, as the education system's performance is often assessed through pupils' and students' educational outcomes, which are primarily assessed through qualitative indicators rather than strictly quantitative metrics. To address this challenge, the researchers identified recurring themes within the reports and included specific pupil and student groups in the measure descriptions to develop well‐defined performance measures. Through this approach, the research group derived 29 measures for the education questionnaire.

### 2.2 Phase 2: Validity Through Actor Input

Face and content validity were studied through two online questionnaires among a wide range of actors from healthcare and education. These participants assessed the most relevant performance measures of (a) healthcare availability and (b) the continuity of education in the Netherlands during a pandemic situation (see Appendix). The questionnaires excluded privacy‐sensitive topics, thus not requiring ethical approval per the Dutch Medical Research Involving Human Subjects Act (Wet Medisch‐Wetenschappelijk Onderzoek met Mensen, 1998).

The questionnaires, structured identically, consisted of closed questions for selecting and ranking the listed variables of healthcare or education and limited additional socio‐demographic professional background questions (see Appendix 1).

#### 2.2.1 Inclusion Criteria and Recruitment

Actors from healthcare and education, including decisionmakers, stakeholders, and experts were invited. Decisionmakers are responsible for making (pandemic‐related) decisions, while stakeholders are affected by these decisions [35, 36]. Experts are researchers, specialists, or advisors in specific fields. Actors were invited through purposeful and snowball sampling by e‐mail or LinkedIn. To ensure a broad representation, actors from several sectors were invited. For healthcare, primary, acute, chronic, and psychiatric care actors were included to represent acute and chronic care. For education, participants from all Dutch educational sectors were included, including primary, secondary, professional, and higher education. Due to affiliations with the University Medical Centre of Nijmegen and Radboud University, selected hospital practitioners and university professors were invited.

#### 2.2.2 Data Collection and Analysis

We used online questionnaires with Qualtrics. Participants were presented with the list of performance measures developed in Phase 1 (see Appendix 1) and were asked to select the five most relevant measures to assess the healthcare availability or educational continuity in the Netherlands, before, during, and after a pandemic. The cut‐off of five measures allows enough information to be gathered in several areas while remaining limited to the essential measures. Subsequently, participants were asked to rank the five measures they had chosen in the previous question from most to least important. A third follow‐up question allowed the participant to add any measures they felt were missing yet relevant. Following these three substantive questions, participants answered three socio‐demographic questions regarding their job, expertise, and job location.

All closed questions were analysed with descriptive statistics. Responses to the open‐ended question were systematically categorized by topic and then aggregated to identify recurring themes in participants' additional comments. Lastly, the socio‐demographic questions helped examine participant expertise and representation in strata in healthcare and education.

## 3 Results

### 3.1 Healthcare Questionnaire

From mid‐June to August 2022, 177 healthcare actors, including 20 identified via snowball sampling, were invited to the questionnaire. Ultimately, 46 actors completed the questionnaire (response rate of 26%). One participant exited the questionnaire after responding to the first question regarding the five most important measures. The participants of the healthcare questionnaire (Table 1) can be divided into two categories: actors with direct patient contact (*n* = 19) and actors in managerial positions (*n* = 20). Alternatively, the group existed of 17 experts, 15 decisionmakers, 13 stakeholders, and 1 unknown. Most participants work in Gelderland‐Zuid (*n* = 21), while 11 participants work in more than one region of the Netherlands.

**TABLE 1:** Demographic data (current profession and workplace) of participants of the healthcare questionnaire.

Demographic data healthcare participants	N
Number of participants	46
Current profession	
Management	11
Medical specialist	12
Policy advisor	5
General practitioner	5
Director	3
Researcher	3
Professor	3
Policy officer	1
Nurse practitioner	1
Epidemiologist	1
Unknown	1
Region of work	
Gelderland‐Zuid	21
Outside Gelderland‐Zuid	13
2 regions	6
> 2 regions	5
Unknown	1

In the healthcare questionnaire, participants chose the top five out of 25 performance measures to indicate the resilience of the healthcare system. Table 2 displays the number and percentage of participants who selected each measure as the top five important, and the frequency of measures ranked as most important. The five measures indicated most often as one of the five most relevant measures were:

1. Pressure on follow‐up care, defined as the number of patients waiting for follow‐up care by geriatric rehabilitation, access to general practitioners, allied health professionals, and district nursing facilities (*n* = 28)
2. Pressure on hospital care (score 1‐5), defined by whether and how the quality and safety of hospital care is safeguarded (*n* = 23)
  1. Manageable, routine internal measures and regular agreements suffice
  2. Concerning, regular buffer capacity is fully depleted, but no special measures are required yet
  3. Serious, special measures and shifts in focus are necessary to maintain quality
  4. Critical, adaptive capacity is fully depleted, putting healthcare quality at risk
  5. Unmanageable, Healthcare quality and/or safety can no longer be guaranteed
3. Absenteeism within healthcare, defined as the short‐term and long‐term absence by sick leave of healthcare workers (*n* = 18)
4. Waiting time per type of care (emergency care, critical plannable intensive care, and plannable intensive and regular hospital care) (*n* = 18)
5. The percentage of hospitals that downscale critical plannable care, defined as care that needs to be delivered within 1–6 weeks (*n* = 16)

**TABLE 2:** Number and percentage of healthcare participants voting for each performance measure of healthcare.

Performance measures of healthcare	N of votes per measure (%)	% of participants per measure (*n* = 46)	N of votes with highest rank (*n* = 45)
Pressure on follow‐up care, defined as the number of patients waiting for follow‐up care by geriatric rehabilitation, access to general practitioners, allied health professionals, and district nursing facilities	28 (12%)	60.9%	4
Pressure on the hospital care (score 1‐5), defined by whether and how the quality and safety of hospital care is safeguarded	23 (10%)	50.0%	11
Absenteeism within healthcare, defined as the short‐term and long‐term absence by sick leave of healthcare workers	18 (8%)	39.1%	3
Waiting time per type of care (emergency care, critical plannable intensive care, and plannable intensive and regular hospital care)	18 (8%)	39.1%	5
The percentage of hospitals that downscale critical plannable care, defined as care that needs to be delivered within 6 weeks	16 (7%)	34.8%	2
Pressure on the ICU (score 1‐5), defined by whether and how the quality and safety of clinical care are safeguarded	14 (6%)	30.4%	3
Availability of ICU capacity	13 (6%)	28.3%	3
Waiting time (in weeks) after registration for mental healthcare	11 (5%)	23.9%	0
Waiting time per healthcare sector	10 (4%)	21.7%	2
The percentage of hospitals scaling down urgent care, defined as care that needs to be delivered within 1 week	10 (4%)	21.7%	4
The percentage of hospitals scaling down plannable care, defined as care that can be delayed by 6 weeks	10 (4%)	21.7%	2
Waiting time per medical specialty	9 (4%)	19.6%	2
The number of postponed surgeries, defined as the increase in the number of regular working weeks and number of surgeries per week	9 (4%)	19.6%	1
The percentage of occupied long‐term care beds per healthcare sector	8 (4%)	17.4%	0
The number of postponed surgeries by specialty, defined as the increase in the number of regular working weeks and number of surgeries per week	8 (4%)	17.4%	1
Total number of surgeries (invasive and intensive procedures) performed weekly	5 (2%)	10.9%	0
Waiting time per type of consultation (treatment, diagnosis, or outpatient visit)	4 (2%)	8.7%	0
Total number of patients in care per week in hospitals (minimally outpatient contact)	3 (1%)	6.5%	0
The number of non‐urgent surgeries performed weekly, defined as care that can be delayed by 6 weeks	3 (1%)	6.5%	0
The percentage of closed operating rooms	2 (1%)	4.3%	0
The national number of referrals to hospitals and independent treatment centres	2 (1%)	4.3%	0
The number of urgent surgeries performed weekly, defined as care that needs to be delivered within 1 week	2 (1%)	4.3%	0
The number of plannable surgeries performed weekly, defined as care that needs to be delivered within 6 weeks	2 (1%)	4.3%	1
Number of referrals of adolescents to mental healthcare per week	2 (1%)	4.3%	0
The number of new diagnoses per specialty	1 (0%)	2.2%	1

Though ‘pressure on follow‐up care’ was chosen most often (28 votes), ‘pressure on the hospital care (score 1‐5)’ was indicated to be the most important by 11 participants (Table 2, column 4). With 11 votes, ‘pressure on the hospital care’ remains distinct among the top 5 relevant measures. Twenty‐five participants, or 56%, ranked one of the five most‐voted measures as their most important.

When asked if measures were missing, 20 participants responded. Five participants proposed primary care measures, such as the number of General Practitioner consultations or the average waiting time to see a General Practitioner, two suggested emergency department measures, and four on personnel absenteeism. Individual participants additionally suggested: personnel and stock capacity (e.g., scalable bed numbers, patient‐to‐nurse ratios, materials available), pandemic‐specific data (e.g., number of hospitalizations due to the pandemic), long‐term effects (e.g., number of late‐stage diagnoses, number of deaths from postponed care, number of unperformed preventive measures), and number of declared medical acts, including preventive actions and processing times.

### 3.2 Education Questionnaire

Two hundred forty‐four actors were invited to partake in the education questionnaire between August and October 2022. Twenty of these actors were contacted through snowball sampling. The participants were asked to define the most critical measures for the continuity of education in the Netherlands. Of the 101 actors who accessed the questionnaire, 71 started (response rate 29%), of which 67 participants completed the questionnaire. The four participants who partially filled in the questionnaire stopped after choosing the five most relevant measures, but before ranking these. This group consisted of (Table 3) 28 stakeholders, 23 experts, 11 decisionmakers, and 9 unknown. Most participants (*n* = 52) work within higher educational institutions and are active within the region of Gelderland (*n* = 51). Most participants are lecturers (*n* = 38), 12 participants hold managerial positions, and three in policymaking positions.

**TABLE 3:** Demographic data (current profession, educational sector, and workplace) of participants of the education questionnaire.

Demographic data education participants	N
Number of participants	71
Current profession	
University lecturer	18
Lecturer	13
Manager	10
Unknown	9
Medical specialist	7
Professor	7
Coordinator educational programme	2
Policy advisor	2
Policy officer	1
Psychologist	1
Internal mentor	1
Educational sector	
Higher education	52
Primary school	6
Secondary education	6
Secondary and vocational education	1
Multiple sectors	2
Unknown	4
Region of work	
Gelderland	51
Outside Gelderland‐Zuid	14
2 regions	1
> 2 regions	1
Unknown	4

Table 4 shows the measures ranked by the participants. Starting with 29 measures, participants identified the following five as the most relevant educational system performance measures:

1. Motivation, well‐being, and the social‐emotional development of pupils (*n* = 41)
2. Psychological problems of pupils and students (*n* = 33)
3. Work pressure among teaching staff (*n* = 32)
4. Quality and intensity of remote learning (*n* = 32)
5. Study delay in higher education (*n* = 28)

**TABLE 4:** Number and percentage of education participants' voting for each performance measure of education.

Performance measures of education	N of votes per measure (%)	% of participants per measure (*n* = 71)	N of votes with highest rank (*n* = 68)
Motivation, well‐being, and the social‐emotional development of pupils	41 (12%)	57.7%	20
Psychological problems of pupils and students	33 (9%)	46.5%	7
Work pressure among teaching staff	32 (9%)	45.1%	4
Quality and intensity of remote learning	32 (9%)	45.1%	4
Study delay in higher education	28 (8%)	39.4%	5
Absenteeism of staff due to COVID‐19‐related problems	20 (6%)	28.2%	1
Learning delay in primary education	18 (5%)	25.4%	6
Learning delay in secondary education	17 (5%)	23.9%	0
Learning delay of pupils by parental education (low, middle, and higher education)	15 (4%)	21.1%	4
Learning delay of pupils by parental income (low, middle, and high income)	14 (4%)	19.7%	3
Pupil absence due to COVID‐19‐related problems	12 (3%)	16.9%	0
Dropout of students in higher education	10 (3%)	14.1%	1
Number of classes sent home due to COVID‐19‐related problems	10 (3%)	14.1%	5
Student learning delay by parental migration background (western, non‐western)	9 (3%)	12.7%	0
Study delay students in secondary vocational education	8 (2%)	11.3%	0
Teaching time in schools (relative to regular teaching time)	8 (2%)	11.3%	1
Number of schools providing full in‐person education in primary education	6 (2%)	8.5%	1
Number of schools providing full in‐person education in secondary education	5 (1%)	7.0%	1
Number of schools providing full in‐person education in secondary vocational education	5 (1%)	7.0%	2
Number of schools providing full in‐person education in higher education	5 (1%)	7.0%	1
Dropouts in primary education	4 (1%)	5.6%	0
Dropout of students in secondary vocational education	4 (1%)	5.6%	1
Dropout from exams	4 (1%)	5.6%	0
Learning delay in schools by percentage of pupils with low‐educated parents	4 (1%)	5.6%	1
Supply of extra teaching time in addition to regular teaching times	4 (1%)	5.6%	0
Grade retention in secondary education	3 (1%)	4.2%	0
Learning delay in schools by school size	3 (1%)	4.2%	0
Learning delay in schools by degree of urbanisation	1 (0%)	1.4%	0
Learning delay of pupils by family composition (two parents, one parent)	0 (0%)	0.0%	0

The measure ‘motivation, well‐being, and the social‐emotional development of pupils’ was indicated most frequently (57.7%) and considered the most important among the top 5 measures. The top five measures were also considered the most important for 40 (59%) of the participants.

Twenty‐four participants gave additional recommendations regarding the list of performance measures. Six participants reported grades, study delay, or success rate of students as important measures. Six participants proposed measures for the teaching staff's quality and skills (e.g., flexibility of teaching staff and capability for online teaching). Five recommendations focused on social aspects (e.g., sense of school community, quality of online student‐teacher relationship, and the number of student interactions online and in‐person). Two responses concerned the ratios of live, online, and/or cancelled teaching hours. Single participants had diverse suggestions: one proposed objective, unbiased metrics, while another argued for broad, non‐sector‐specific measures. Additionally, one participant opposed measures targeting specific strata, another suggested that measures addressing distinct student characteristics could provide richer data.

## 4 Discussion

This study adapted an existing approach to identify potentially useful performance measures of resilience in healthcare and education systems during a pandemic. While the findings provide valuable insights, they are based on a limited number of Dutch actors, and further research is needed to assess their applicability across different contexts and societal systems to inform policy decisions. However, the method can be applied internationally, allowing others to develop their own indicators within the COPEWELL framework rather than directly adopting the ones identified in this study. A questionnaire of actors identified key performance measures: in healthcare, these include care demand, care quality, staff absenteeism, waiting times, and care downscaling; in education, the focus was on pupil development, student psychology, teacher workload, remote learning effectiveness, and higher education delays.

Adopting the COPEWELL model proved a feasible method, which can result in a few essential resilience performance measures with face and content validity. Face validity stems from the preliminary list of performance measures based on trustworthy sources and expertise within the research group (medical specialists and professors), while content validity is supported by participants linking the measures to resilience and providing additional feedback. This participatory development of performance measures can be executed in a short time period. Consequently, such measures can be developed and used to monitor disaster or pandemic effects on societal functioning, thereby assisting policymaking.

### 4.1 The Final Set of Performance Measures

The questionnaire assessed the most relevant performance measures for measuring resilience within the healthcare and education system. In principle, this may lead to overarching performance measures for the prioritised societal system sectors during a pandemic.

For healthcare, three of the final five performance measures comprise hospital care. Only one performance measure can truly be considered applicable to the whole healthcare system. Based on the Dutch national registries, the initial list of performance measures focused mainly on acute healthcare. This overrepresentation of hospital care with an underrepresentation of, for example, primary care, was highlighted in the responses to the open question. A few participants remarked in the questionnaire that they would have liked to have included more primary care measures, suggesting a potential gap. The final five healthcare performance measures reflect an emphasis on acute care, mirroring the national policy focus in the Netherlands throughout the COVID‐19 pandemic. Four of the final healthcare performance measures used interval scales, with only one—pressure on hospital care—measured categorically.

For education, the performance measures were more overarching and interspersed between different educational sectors, with three of the five measures applicable to all educational sectors (primary, secondary, and higher education). This suggests that resilience in education may be assessed through overarching indicators rather than sector‐specific ones. Despite the overrepresentation of higher education actors in the sample, the chosen measures still captured system‐wide concerns. Unlike healthcare, four of the education performance measures were categorical rather than interval‐based, possibly indicating challenges in quantifying these aspects. This may be due to the original formulation of indicators requiring further research to quantify these measures. This could also mean that some performance measures are more easily standardised across different contexts, potentially allowing the same measure to be applied across educational sectors while stratifying results for sector‐specific insights.

Comparing the two systems, a key distinction is the sectoral scope of the performance measures. While healthcare measures were heavily weighted towards acute care, education measures were more universally applicable across different levels of the system. In both sets of performance measures, staff well‐being was represented, underlining its crucial role in system functioning. Additionally, the difference in measurement types—predominantly interval scales in healthcare versus categorical measures in education—suggests that healthcare indicators may be more readily standardized, whereas education measures may require further refinement for broader applicability.

### 4.2 Comparison of Findings With Existing Resilience Indicators for Healthcare and Education

#### 4.2.1 Healthcare

Results indicated pressure on follow‐up care, pressure on hospital care, absenteeism of healthcare staff, waiting times, and downscaling critical plannable care as the five most important performance measures of healthcare during a pandemic. The COPEWELL rubric for community assessment specifies health and well‐being as one of four domains [37]. Currently, these rely on County health rankings data [38, 39], which assess long‐term health and social factors to support recovery after disasters [39]. However, an ongoing pandemic requires dynamic resilience indicators that capture short‐term dynamics rather than long‐term trends.

Fleming et al. reviewed empirical research on indicators to assess health system resilience against shocks [40]. They found four indicator areas: resources, service delivery, governance, and finance. While this review found healthcare resources to be the most frequent indicator in 68 studies, only one of our performance measures, absenteeism of healthcare workers, can be classified in this group. However, the second most frequent group of indicators, service delivery, encompasses our questionnaire's remaining four performance measures: follow‐up care, pressure on hospital care, waiting times, and downscaling critical plannable care. Reflecting upon our initial set of performance measures, it is notable that they only focused on service delivery and resources, without including performance measures related to governance or financing. We focused on Dutch national registries, revealing a strong emphasis on acute service delivery and resources during the COVID‐19 pandemic. In contrast, Flemings' paper included not only pandemics but also economic crises, natural disasters, and war as crises impacting healthcare system resilience.

The differences in findings between our questionnaire, COPEWELL, and Fleming et al. may be that our questionnaire did not include other categories, such as governance and finances. Participants were instructed to focus on performance measures for accessibility of healthcare services, placing a strong focus on healthcare resources. Another reason could be that this proved to be the area where the acute needs were highest during the pandemic.

#### 4.2.2 Education

One of the four domains in the COPEWELL rubric for community resilience specifies education under life necessities. In 2017, COPEWELL defined indicators of education resilience as the capacity to provide stable education with open schools, pupil‐to‐teacher ratios in public schools, and the percentage of ninth‐graders graduating within four years [16]. Compared to this, our survey measures encompass learning delay, remote learning, well‐being of students, and work pressure of staff.

Due to the COVID‐19 pandemic, the EU has initiated national resilience and recovery plans through the Next Generation EU (NGEU) project to mitigate the socio‐economic impact of the pandemic [41]. The EU monitors three education indicators biannually for member states: (i) the percentage of the population participating in education or training, (ii) the percentage of the population using new or modernised childcare and education facilities, and (iii) number of young people (age 15‐29) receiving support through the NGEU project. In contrast to our questionnaire, EU‐indicators do not assess student or pupil well‐being, staff work pressure, and remote learning quality. These NGEU indicators are formulated as European goals aimed to enhance resilience and accelerate recovery in future crises, including specifically for healthcare and education.

### 4.3 The Pace of Pandemic Impact on Healthcare and Education Resilience

Education and healthcare are very different systems when it comes to their resilience. Healthcare feels pandemic consequences sooner than the educational system, where the effects are observed later. The prioritised performance measures illustrate this difference. Healthcare measures can be measured more directly, while education measures cover a longer time span and are more categorical. For example, ‘pressure on hospital care’ is measured in real‐time, while ‘motivation, well‐being, and the social‐emotional development of pupils’ require time before measurable shifts occur. This difference between the healthcare and education systems has frequently caused policymakers to prioritise healthcare to the detriment of educational system because the effects could not be measured yet [42]. Studies are gradually showing COVID‐19 measures' impact on students throughout the academic sectors. For instance, Buffel's’ study shows a correlation between workplace closure and stay‐at‐home restrictions on depressive symptoms in higher education students [43].

### 4.4 Development of Performance Measures of Resilience

Previous research has described various methods to develop resilience indicators without a standard or common practice, considering the broad scope in which resilience indicators are applied. Many papers remain brief in describing the development of resilience indicators and instead focus on the outcome of finalised resilience indicators. On the other hand, the COPEWELL method provides a conceptual framework to model a system's resilience to a disaster and uses a standard to develop resilience indicators [16, 18]. It offers an extensive and comprehensive framework for resilience to disasters that improves the understanding of resilience amongst involved actors and policymakers [44]. The COPEWELL framework strongly focuses on the United States and only briefly discusses the development of resilience indicators.

Our work suggests that the COPEWELL framework has potential applicability to the COVID‐19 pandemic in the Netherlands. This method to develop performance measures could be used for each of the nine essential systems in a disaster defined by COPEWELL (communication, economy, education, food and water, government, housing, healthcare and public health, nurturing and care, transportation, and well‐being) to identify crucial measures within each system. However, further research is needed to systematically validate the applicability of the final performance measures and to assess the framework's value in real‐world policy and decision‐making contexts.

#### 4.4.1 Strengths and Limitations

While a key strength of this study is that it is based on a well‐developed and validated disaster management system, which was adapted to the pandemic context, we have not fully implemented the COPEWELL co‐development process. The digital questionnaire we used enabled us to contact actors quickly and broadly during a pandemic. The response rates of 26% in healthcare and 29% in education participants align with the anticipated response rate for online questionnaires [21].

A limitation is that the sample groups may not be fully representative of each sector in the system, with an overrepresentation of certain sectors. Despite an overrepresentation of higher education the final education performance measures did not convey this. Overrepresentation of (acute) hospital care and underrepresentation of, for example, primary care, reflects pandemic policymaking in the Netherlands. Participants were also dominantly based in Gelderland‐Zuid, making the generalisation to the Netherlands debatable. However, the pandemic context in Gelderland‐Zuid parallelled the overall trends in the Netherlands. While participant demographics may influence the selected performance indicators, multivariate analysis was not feasible due to data limitations and requires further exploration. As an exploratory step, this study prioritised the identification of the most relevant indicators, minimising participant burden, and optimising response rates. Similarly, gender data were not collected, as the study aimed to identify potential resilience indicators rather than individual preference differences. As a result, a gender‐based analysis was not possible, but future research could explore this aspect.

Lastly, further clarification and sub‐stratum definitions of the final performance measures and clarity on how to quantify and measure them, is necessary should they be used to navigate decision making in the future.

### 4.5 COPEWELL and Recommendations for future Research

This article focused on phases 1 and two of the COPEWELL Rubric, which suggests two more phases (see Figure 1). Future work can extend our workphases by pilot testing the developed performance measures and developing a computational monitoring system.

#### 4.5.1 Phase 3: Pilot Test Performance Measures

The developed performance measures should be described in specific variables to monitor resilience over time. This can be obtained by calculating dynamic indicators of resilience (DIORs) from the collected data [45, 46]. This requires time series analysis showing changes in resilience over time which can guide policymakers in their pandemic management.

#### 4.5.2 Phase 4: Monitoring System

This study looked at performance measures of systems separately from one another. The next step is to look at the performance measures of these systems in relation to one another. As the COPEWELL model suggests, computational modelling should be the next step for real‐time resilience [16]. Next, simulating policymaking with such models can enhance policymakers' awareness and understanding of the impact of different policy measures and most‐importantly exemplify (deep) uncertainty in these outcomes. Computational models of connected systems such as healthcare and education may deliver scenarios on how policy decisions in one system might affect another system. This would enable policymakers to envision possible unintended consequences and design interventions that mitigate them. Consequently, policy measures could be taken in other systems that could spare the healthcare system from extra stressors during a pandemic. As such, the availability of performance and resilience measures tailored to capture crucial societal functions in a pandemic has also been recommended by other researchers.

Saulnier et al. point out the need for connections between societal and healthcare system resilience [8]. If applied, future research should evaluate the added value of these DIORs, and the computational models.

## 5 Conclusion

This study demonstrated that it is feasible to develop performance measures for assessing the resilience of the healthcare and education systems in a pandemic context. Using the COPEWELL framework as a guiding principle, we identified key performance measures through expert input, ensuring face and content validity. However, the findings are based on a sample of Dutch actors, with a strong representation from higher education and acute healthcare, which may not fully capture the resilience needs of other sectors, such as primary care and vocational education. While the identified performance measures provide insight into systemic resilience, their applicability in real‐world policy decision‐making requires further validation. Future research should explore the generalisability to different contexts and can elaborate upon this by using the expert‐chosen performance measures of resilience in time‐series data to analyse and recognise trends in resilience for policymaking. Subsequently, the added value of using resilience indicators can be evaluated. An annotated computational model illustrating the variables impacting the DIORs across societal domains could further inform policymaking. For pandemic monitoring, we strongly recommend that resilience be linked across different societal systems to form a globally coherent understanding of societal resilience in pandemics. This will broaden the applicability of resilience measures for pandemic management, making policymakers better equipped to assess and strengthen system resilience in future pandemics.

## Conflicts of Interest

The authors declare no conflicts of interest.

## Data Availability

The data that support the findings of this study are available from the corresponding author [SH], upon reasonable request.

## Appendix 1

### Availability of Healthcare Questionnaire

#### Introduction

##### PanAdapt Project

The PanAdapt project researches decision making in pandemics, such as the COVID‐19 pandemic, and its impact on healthcare and society. Effective decision making requires, among other things, consideration of the resilience of the healthcare system. Here, resilience is the ability of the healthcare system to adapt to new difficult circumstances (such as the high COVID pressure in hospitals) so that it can continue to function well and serve society adequately.

##### Purpose of This Questionnaire

The purpose of this questionnaire is to gain insight into indicators for measuring the availability of healthcare in the Netherlands before, during and after a pandemic. By gaining insight into changes in this availability, and thus the functioning of the healthcare system, insight can be gained into the resilience of the Dutch healthcare system, among other things. At a later stage of this research, the results of this questionnaire will be presented to decisionmakers in order to eventually learn to better deal with the uncertainty of pandemic management and to support them in developing policies to make healthcare and society more resilient.

Click on the right arrow to proceed to the general instructions of the questionnaire.

#### General Instructions Questionnaire

This questionnaire is about healthcare availability and consists of three parts (A, B and C). In part A, we ask about indicators of healthcare availability. In section B we ask some demographic data (anonymously) such as your area of expertise. In section C, we ask if you know any other experts in the field of healthcare to participate in this survey. The questionnaire takes about 7 min to complete.

We ask that you read the questions carefully. They will tell you how to answer the question and what the question relates to. It is important for the success of this survey that you do not skip any of the questions.

If you have any questions or comments about the questionnaire, please contact XXX.

Click on the right arrow to begin the questionnaire.

#### Part A: Indicators of Availability in Healthcare

**A1.** The following is a list of indicators of availability in healthcare. The indicators come from the Dutch Healthcare Authority's “Monitor Accessibility of Care ‐ December 24, 2021”. In this report, the Dutch Healthcare Authority reports on the impact of COVID‐19 on the accessibility of care in the Netherlands.

We want to know which indicators from the list below you consider most relevant to determine the availability of healthcare in the Netherlands. Please go through the entire list carefully and then indicate which 5 indicators you would include to measure healthcare availability before, during and after a pandemic. **In other words, provide 5 answers.**

□ Pressure on follow‐up care (number of patients waiting for geriatric rehabilitation, family medicine, paramedic and district nursing care)
□ Pressure on clinic (1–5). 1 = Use internal measures and regular appointments are sufficient (manageable). 5 = Quality and/or safety of care can no longer be guaranteed (unmanageable)
□ Sick leave within care (short‐term (1/tm 91 days) and long‐term (92–730 days)),
□ Waiting time by type of care (urgent care, critically plannable care and plannable care)
□ The percentage of hospitals scaling down in critical plannable care (Severity Class 3)
□ ICU pressure (1–5). 1 = Use internal measures and regular appointments are sufficient (manageable). 5 = Quality and/or safety of care can no longer be guaranteed (unmanageable)
□ Availability of ICU capacity.
□ Enrolment waiting times (in weeks) for the mental health sector (independent and institutional)
□ Waiting time by healthcare sector
□ The percentage of hospitals scaling down in urgent care (Severity Classes 1 and 2)
□ The percentage of hospitals scaling down in planned care (Severity Classes 4 and 5)
□ Waiting time per specialty
□ The number of deferred surgeries (increase in number of regular working weeks and number of surgeries per week)
□ The percentage of occupied long‐term care beds by healthcare sector
□ The number of deferred surgeries by specialty (increased number of regular working weeks and number of surgeries per week)
□ Total number of surgeries (invasive and intensive procedures) performed weekly
□ Waiting time by type of consultation (treatment, diagnosis, or outpatient visit)
□ Total number of patients in care per week in hospitals (minimum outpatient contact)
□ The number of non‐urgent surgeries (Severity Classes 4 and 5) performed weekly
□ The percentage of closed operating rooms
□ The national number of referrals to hospitals and independent treatment centres (ZBC)
□ The number of urgent surgeries (Severity Classes 1 and 2) performed weekly
□ The number of schedulable surgeries (Severity Class 3) performed weekly
□ Number of adolescent referrals to the mental health system per week
□ The number of new diagnoses per specialty

**A2.** On the left you will see the five indicators you indicated as the most important to measure healthcare availability before, during and after a pandemic. Not quite sure yet? You can change your selection by going back to the previous question (via left arrow, bottom left).

Drag your selected indicators from the left column to the right column and rank from most important to least important. Place the most important indicators at the top. Place the less important indicators at the bottom. You can change the order in the right column at any time by dragging the indicators up or down.

Do you agree with the ranking? Then press the right arrow to continue with the questionnaire.

**A3.** Are there any indicators that you feel are highly relevant to determining availability in healthcare but were missing from the list? Please note them here.

#### Part C: Other Experts

We are looking for participants.

For this questionnaire, we are looking for as many healthcare experts as possible. These may be experts from different fields of healthcare. Parallel to this questionnaire we also have a questionnaire for resilience of the education system in the Netherlands before, during and after a pandemic. We are therefore also looking for experts within the education system.

In the next stage of our research, we are looking for policymakers within the healthcare system and across multiple domains (e.g., healthcare and education). In this way we want to investigate how policymakers deal with the indicators established by experts from each domain.

If you have any questions, please contact XXX.

**C1.** Do you know experts within the healthcare domain who can or should estimate the resilience of the healthcare system? Please list here name and e‐mail address and/or institution where this person works. We will invite them, without obligation, to complete the questionnaire as well.

**C2.** Do you know experts within the field of education who can or should estimate the resilience of the education system? Please list here name and e‐mail address and/or institution where this person works. We will invite them, without obligation, to participate in our research.

**C3.** Do you know anyone who is a policymaker or administrator in healthcare, or needs to make or prepare policy decisions about healthcare and another domain? Please write their name and e‐mail address and/or the institution where they work. We will invite them, without obligation, to participate in our research.

This is the end of the questionnaire, click the right arrow to complete the questionnaire and submit your answers.

Thank you again for your participation in this questionnaire.

Would you like to follow up on further developments in the PanAdapt project?

You can do so via LinkedIn or Twitter.

Do you have any questions regarding your participation in this questionnaire?

Please contact XXX.

### Continuity of Education Questionnaire

#### Introduction

##### PanAdapt Project

The PanAdapt project conducts research on decision making in pandemics, such as the COVID‐19 pandemic, and the effects of related measures on education, healthcare and other societal domains. Effective decision making requires, among other things, consideration of the resilience of the education sector. Here, resilience is the adaptive capacity of the education sector to minimise the negative impact of the pandemic and drastic measures (such as school closures and class cancellations due to student or teacher absence) on the availability and quality of education.

##### Purpose of This Questionnaire

The purpose of this questionnaire is to gain insight into indicators for measuring the continuity of availability and quality of education in the Netherlands before, during and after a pandemic. By gaining insight into changes in that continuity, insight can be gained into the resilience of the Dutch education system, among other things. At a later stage of this research, the results of this questionnaire will be presented to decisionmakers in order to ultimately learn to better deal with the uncertainty of pandemic management, and to support them in developing policies to make the education system and other societal domains more resilient.

Click on the right arrow to proceed to the general instructions of the questionnaire.

#### General Instructions Questionnaire

This questionnaire is about continuity of education and consists of three parts (A, B and C). In part A, we ask for indicators to determine the continuity of the education system. In section B we ask some demographic data (anonymously) such as your area of expertise. In section C, we ask if you know any other experts in the field of healthcare to participate in this survey. The questionnaire takes about 10 min to complete.

We ask that you read the questions carefully. They will tell you how to answer the question and what the question relates to. It is important for the success of this survey that you do not skip any of the questions.

If you have any questions or comments about the questionnaire, please contact XXX.

Click on the right arrow to begin the questionnaire.

#### Part A: Indicators of Continuity of Education

**A1.** The following is a list of indicators to determine the continuity of education in the Netherlands. The indicators come from reports such as the ‘National Cohort Study on Education’ by the National Regieorgaan Onderwijsonderzoeken, and the ‘State of Education 2021’ by the Inspectorate of Education. Because we are interested in the overall state of the Dutch education system, we have tried to generalise this list as much as possible to all education sectors (primary education, secondary education, special education, secondary vocational education and higher education) at both national and regional levels.

We want to know which indicators from the list below you consider most relevant in order to map the continuity of the Dutch education system. Please go through the entire list carefully and then indicate which 5 indicators you would include to measure the continuity of education before, during and after a pandemic. **In other words, provide 5 answers.**

□ Motivation, well‐being and students' social‐emotional development
□ Psychological problems pupils and students
□ Work pressure among teaching staff
□ Quality and intensity of remote learning
□ Study delays in higher education
□ Staff absences due to COVID‐19‐related problems
□ Learning delay in primary education
□ Learning delay in secondary education
□ Student learning delay by education parents (low medium high)
□ Learning delay of pupils by income parents (low modal high)
□ Pupil absence due to COVID‐19‐related problems
□ Attrition of students in higher education
□ Amount of classes sent home to COVID‐19‐related problems
□ Student learning delay by migration background parents (western, non‐western)
□ Study delay students in secondary vocational education
□ Teaching time schools (relative to regular teaching time)
□ Number of schools providing full physical education in primary education
□ Number of schools providing full physical education in secondary education
□ Number of schools providing full physical education in secondary vocational education
□ Number of schools providing full physical education in higher education
□ Dropouts in primary education
□ Dropout of students in secondary vocational education
□ Dropout from exams
□ Learning delay in schools by percentage of students with low‐educated parents
□ Offering extra teaching time in addition to regular teaching times
□ Dropouts in secondary education
□ Learning delay in schools by school size
□ Learning delay in schools by degree of urbanisation

**A2**. On the left you will see the 5 indicators you indicated as the most important to determine educational continuity before, during and after a pandemic. Not quite sure yet? You can change your selection by going back to the previous question (via left arrow, bottom left)

Drag your selected indicators from the left column to the right column and rank from most important to least important. Place the most important indicators at the top. Place the less important indicators at the bottom. You can change the order in the right column at any time by dragging the indicators up or down.

Do you agree with the ranking? Then press the right arrow to continue with the questionnaire.

#### Part C: Other Experts

We are looking for participants.

For this questionnaire we are looking for as many experts in education as possible. These may be experts from different sectors of the Dutch education system.

In a next stage of our research we are looking for policymakers within education and across multiple domains (e.g. healthcare and education). In this way we want to investigate how policymakers deal with the indicators established by experts from each domain.

If you have any questions, please contact XXX.

**C1.** Do you know any experts within education who can or should estimate the resilience of the education system? Please list here name and e‐mail address and/or institution where this person works. We will invite them, without obligation, to participate in our research.

**C2.** Do you know any policymaker or administrator in education, or in another field such as healthcare, who needs to make or prepare policy decisions? Please list here name and e‐mail address and/or institution where this person works. We will invite them, without obligation, to participate in our research.

This is the end of the questionnaire, click the right arrow to complete the questionnaire and submit your answers.

Thank you again for your participation in this questionnaire.

Would you like to follow up on further developments in the PanAdapt project?

You can do so via LinkedIn or Twitter.

Do you have any questions regarding your participation in this questionnaire?

Please contact XXX.

## References

[1] OECD . COVID‐19 pandemic underlines need to strengthen resilience of health systems, says OECD, (November 2021), https://www.oecd.org/health/covid‐19‐pandemic‐underlines‐need‐to‐strengthen‐resilience‐of‐health‐systems‐says‐oecd.htm. Accessed September 9, 2022.

[2] D. Wernli , M. Clausin , N. Antulov‐Fantulin , et al., “Building a Multisystemic Understanding of Societal Resilience to the COVID‐19 Pandemic,” BMJ Global Health 6, no. 7 (2021): e006794, 10.1136/bmjgh-2021-006794. PMC8300552 34301677

[3] T. M. Schuurman , L. F. Henrichs , N. K. Schuurman , S. Polderdijk , and L. Hornstra , “Learning Loss in Vulnerable Student Populations After the First Covid‐19 School Closure in the Netherlands,” Scandinavian Journal of Educational Research 67, no. 2 (September 2023): 309–326, 10.1080/00313831.2021.2006307.

[4] (NL) IvhOEI. De Staat van het Onderwijs 2023 [The State of Education 2023] . May 2023, https://www.onderwijsinspectie.nl/documenten/rapporten/2023/05/10/rapport‐de‐staat‐van‐het‐onderwijs‐2023. Accessed Dec 12, 2023.

[5] P. Engzell , A. Frey , and M. D. Verhagen , “Learning Loss Due to School Closures During the COVID‐19 Pandemic,” Proceedings of the National Academy of Sciences U S A. 118, no. 17 (April 2021): e2022376118, 10.1073/pnas.2022376118. PMC8092566 33827987

[6] S. de Leeuw , C. Haelermans , M. Jacobs , R. van der Velden , L. van Vugt , and S. van Wetten , “The Role of Family Composition in Students' Learning Growth During the COVID‐19 Pandemic,” Journal of Marriage and Family 85, no. 3 (2023): 807–828, 10.1111/jomf.12912.

[7] C. Haelermans , R. Korthals , M. Jacobs , et al., “Sharp Increase in Inequality in Education in Times of the COVID‐19‐Pandemic,” PLoS One 17, no. 2 (2022): e0261114, 10.1371/journal.pone.0261114. 35108273 PMC8809564

[8] D. D. Saulnier , K. Blanchet , C. Canila , et al., “A Health Systems Resilience Research Agenda: Moving From Concept to Practice,” BMJ Global Health 6, no. 8 (2021): e006779, 10.1136/bmjgh-2021-006779. PMC8344286 34353820

[9] Health system resilience , “The Organization for Economic Cooperation and Development (OECD),”, https://www.oecd.org/en/topics/sub‐issues/health‐system‐resilience.html. Accessed February 12, 2025.

[10] For a healthy Belgium—Health System Performance Assessment . Accessed February 12, 2025, https://www.healthybelgium.be/en/health‐system‐performance‐assessment.

[11] PHSSR launches expert advisory group to strengthen health system sustainability and resilience in the EU . Partnership for Health System Systainability & Resilience. Accessed February 12, 2025, https://www.phssr.org/eu_expertadvisorygroup.

[12] A. Jovanović , P. Klimek , O. Renn , et al., “Assessing Resilience of Healthcare Infrastructure Exposed to COVID‐19: Emerging Risks, Resilience Indicators, Interdependencies and International Standards,” Environ Syst Decis 40, no. 2 (2020): 252–286, 10.1007/s10669-020-09779-8. 32837821 PMC7271643

[13] M. Haghighat , S. M. Mousavi , and M. Jahadi Naeini , “Identifying and Ranking of the Main Organizational Resilience Indicators in the Hospital During the COVID‐19 Pandemic: A Study Using Fuzzy Delphi Technique (FDT) and Fuzzy Analytical Hierarchy Process (FAHP),” Heliyon 10, no. 5 (March 2024): e27241, 10.1016/j.heliyon.2024.e27241. 38449624 PMC10915563

[14] L. Fisher , “Marten Scheffer, Critical Transitions in Nature and Society,” American Journal of Psychology 124, no. 3 (2011): 365–367, 10.5406/amerjpsyc.124.3.0365.

[15] B. Walker , Cs Holling , S. Carpenter , and A. Kinzig , “Resilience, Adaptability and Transformability in Social‐Ecological Systems,” Ecol Soc 9, no. 2 (11/30 2003): 9, 10.5751/ES-00650-090205.

[16] J. M. Links , B. S. Schwartz , S. Lin , et al., “COPEWELL: A Conceptual Framework and System Dynamics Model for Predicting Community Functioning and Resilience After Disasters,” Disaster Medicine and Public Health Preparedness 12, no. 1 (2018): 127–137, 10.1017/dmp.2017.39. 28633681 PMC8743042

[17] Martin‐Breen P. , Anderies J. Resilience: A Literature Review. presented at: Bellagio Initiative; November 2011; Accessed September 12, 2022, https://opendocs.ids.ac.uk/opendocs/bitstream/handle/20.500.12413/3692/Resilience%20A%20Literature%20Review_summary.pdf?isAllowed=y&sequence=2

[18] M. Schoch‐Spana , K. Gill , D. Hosangadi , et al., “The COPEWELL Rubric: A Self‐Assessment Toolkit to Strengthen Community Resilience to Disasters,” International Journal of Environmental Research and Public Health 16, no. 13 (2019): 2372, 10.3390/ijerph16132372. 31277357 PMC6651431

[19] COPEWELL Communities . John Hopkins University. December 2022. Accessed December 27, 2022, https://www.copewellmodel.org/in‐practice/COPEWELL‐communities.html

[20] A. Snyder , S. Matthew , N. Leahy , et al., “Island Communities and Disaster Resilience: Applying the EnRiCH Community Resilience Framework,” Public Health Nursing 39, no. 1 (2022): 62–70, 10.1111/phn.13007. 34735033

[21] D. D. Nulty , “The Adequacy of Response Rates to Online and Paper Surveys: What Can Be Done?,” Assessment & Evaluation in Higher Education 33, no. 3 (2008/06/01 2008): 301–314, 10.1080/02602930701293231.

[22] M. Mohseni , S. Ahmadi , S. Azami‐Aghdash , et al., “Challenges of Routine Diabetes Care During COVID‐19 Era: A Systematic Search and Narrative Review,” Primary Care Diabetes 15, no. 6 (2021/12/01 2021): 918–922, 10.1016/j.pcd.2021.07.017. PMC8326007 34393092

[23] H. Merrick , H. Driver , C. Main , et al., “Impacts of Health Care Service Changes Implemented Due to COVID‐19 on Children and Young People With Long‐Term Disability: A Mapping Review,” Developmental Medicine and Child Neurology 65, no. 7 (2023/07/01 2023): 885–899, 10.1111/dmcn.15503. 36649197

[24] A. Sud , M. E. Jones , J. Broggio , et al., “Collateral Damage: The Impact on Outcomes From Cancer Surgery of the COVID‐19 Pandemic,” Annals of Oncology 31, no. 8 (2020/08/01/2020): 1065–1074, 10.1016/j.annonc.2020.05.009. PMC7237184 32442581

[25] S. Hammerstein , C. König , T. Dreisörner , and A. Frey , “Effects of COVID‐19‐Related School Closures on Student Achievement‐A Systematic Review. Systematic Review,” Frontiers in Psychology (September 2021): 12, 10.3389/fpsyg.2021.746289. PMC8481663 34603162

[26] E. Panagouli , A. Stavridou , C. Savvidi , et al., “School Performance Among Children and Adolescents During COVID‐19 Pandemic: A Systematic Review,” Children 8, no. 12 (2021): 1134, 10.3390/children8121134. 34943330 PMC8700572

[27] S. Chaabane , S. Doraiswamy , K. Chaabna , R. Mamtani , and S. Cheema , “The Impact of COVID‐19 School Closure on Child and Adolescent Health: A Rapid Systematic Review,” Children 8, no. 5 (2021): 415, 10.3390/children8050415. 34069468 PMC8159143

[28] R. Viner , S. Russell , R. Saulle , et al., “School Closures During Social Lockdown and Mental Health, Health Behaviors, and Well‐Being Among Children and Adolescents During the First COVID‐19 Wave: A Systematic Review,” JAMA Pediatrics 176, no. 4 (2022): 400–409, 10.1001/jamapediatrics.2021.5840. 35040870

[29] J. A. Elharake , F. Akbar , A. A. Malik , W. Gilliam , and S. B. Omer , “Mental Health Impact of COVID‐19 Among Children and College Students: A Systematic Review,” Child Psychiatry and Human Development 54, no. 3 (2023/06/01 2023): 913–925, 10.1007/s10578-021-01297-1. PMC8747859 35013847

[30] 30. Health at a Glance: Europe 2020: State of Health in the EU Cycle . 2020.

[31] A. Y. Björnberg Ap , Euro Health Consumer Index 2018, (February 2019): 25, 90, https://santesecu.public.lu/dam‐assets/fr/publications/e/euro‐health‐consumer‐index‐2018/euro‐health‐consumer‐index‐2018.pdf.

[32] Monitor Toegankelijkheid van Zorg—24 december 2021 . “Nederlandse Zorgautoriteit,” December 2023, https://puc.overheid.nl/doc/PUC_697448_22/1. Accessed September 12, 2022.

[33] De Staat van het Onderwijs 2022 [The State of Education 2022] . “Inspectie Van Het Onderwijs [Education Inspectorate (NL)],”: Updated Apr 13, https://www.onderwijsinspectie.nl/documenten/rapporten/2022/04/13/de‐staat‐van‐het‐onderwijs‐2022.

[34] O. Ivh , COVID‐19‐monitor Inspectie van het Onderwijs meting 3: Hoe hebben scholen en instellingen het onderwijs vormgegeven in de periode vanaf de zomervakantie tot aan begin oktober 2020?, (November 2020), https://www.google.com/url?sa=t&rct=j&q=&esrc=s&source=web=2ahUKEwjJnrf0‐qWEAxWExQIHHUwKDOMQFnoECA0QAw&url=https%3A%2F%2Fwww.onderwijsinspectie.nl%2Fbinaries%2Fonderwijsinspectie%2Fdocumenten%2Fp&cd=&vedublicaties%2F2020%2F11%2F24%2Fcovid‐19‐monitor‐ho‐derde‐meting%2FCOVID‐19‐monitor%2Bmeting%2B3‐Hoger%2BOnderwijs.pdf&usg=AOvVaw2HeqstrJllhd16MuigN7M6&opi=89978449. Accessed September 12, 2022.

[35] R. E. Freeman , Strategic Management: A Stakeholder Approach (Cambridge University Press, 2010).

[36] C. Williams and L. Fang , “A Value‐Focused Multiple Participant‐Multiple Criteria (MPMC) Decision Support Approach for Public Policy Formulation,” Group Decision and Negotiation 28, no. 1 (2019): 99–126, 10.1007/s10726-018-9597-3.

[37] COPEWELL Community Functioning . “Rubric Implementation Guide and Sample Handouts for Self‐Assessment and Action COPEWELL,”, https://copewellmodel.org/sites/default/files/2023‐01/copewell‐communityfunctioning‐implementationguide.pdf. Accessed 30 July, 2023.

[38] John Hopkins COPEWELL model . “Measures Summary Sheet,” COPEWELL. Accessed 31 July, 2023, https://copewellmodel.org/sites/default/files/2023‐01/copewell‐summaryof‐measures.pdf.

[39] Explore health rankings ‐ Alabama . “County Health Rankings & Roadmaps,”, https://www.countyhealthrankings.org/explore‐health‐rankings/alabama?year=2023. Accessed 31 July, 2023.

[40] P. Fleming , C. O'Donoghue , A. Almirall‐Sanchez , et al., “Metrics and Indicators Used to Assess Health System Resilience in Response to Shocks to Health Systems in High Income Countries—A Systematic Review,” Health Policy 126, no. 12 (2022/12/01/2022): 1195–1205, 10.1016/j.healthpol.2022.10.001. PMC9556803 36257867

[41] Education policy in the National Recovery and Resilience Plans . European Parliamentary Research Service, (2022).

[42] D. Gurdasani , C. Pagel , M. McKee , et al., “Covid‐19 in the UK: Policy on Children and Schools,” BMJ 378 (2022): e071234, 10.1136/bmj-2022-071234.

[43] S. Van de Velde , V. Buffel , C. van der Heijde , et al., “Depressive Symptoms in Higher Education Students During the First Wave of the COVID‐19 Pandemic. An Examination of the Association With Various Social Risk Factors Across Multiple High‐ and Middle‐Income Countries,” SSM ‐ Population Health 16 (2021/12/01/2021): 100936, 10.1016/j.ssmph.2021.100936. PMC8484180 34611543

[44] C. C. Slemp , S. Sisco , M. C. Jean , et al., “Applying an Innovative Model of Disaster Resilience at the Neighborhood Level: The COPEWELL New York City Experience,” Public Health Reports 135, no. 5 (2020): 565–570: 2020/09/01, 10.1177/0033354920938012. 32735159 PMC7485050

[45] S. M. W. Gijzel , I. A. van de Leemput , M. Scheffer , M. Roppolo , M. G. M. Olde Rikkert , and R. J. F. Melis , “Dynamical Resilience Indicators in Time Series of Self‐Rated Health Correspond to Frailty Levels in Older Adults,” J Gerontol A Biol Sci Med Sci 72, no. 7 (July 2017): 991–996, 10.1093/gerona/glx065. 28475664

[46] M. Scheffer , J. Bascompte , W. A. Brock , et al., “Early‐warning Signals for Critical Transitions,” Nature 461, no. 7260 (2009): 53–59: 2009/09/01, 10.1038/nature08227. 19727193

